# Exploring Digital Solutions: A Pilot Study on the Intersection of Autistic Traits and Mental Health

**DOI:** 10.1192/j.eurpsy.2025.799

**Published:** 2025-08-26

**Authors:** D. Sönmez, E. B. Yıldız, T. R. Jordan

**Affiliations:** 1 Psychology, Ibn Haldun University; 2 Psychology, İstanbul Bilgi University, İstanbul, Türkiye

## Abstract

**Introduction:**

Digital interventions are increasingly popular in addressing various issues, including mental health problems, executive functions, and social cognitive skills in clinical and non-clinical populations. Previous studies have revealed that neurotypical individuals with elevated autistic traits may be more susceptible to other mental health conditions such as anxiety disorders, mood disorders, internet addiction, substance abuse, and suicidal risk. While there is no definitive explanation for this susceptibility, social and cognitive impairments may be contributing factors, including problems with executive functions and social cognitive skills.

**Objectives:**

The main goal of this research is to develop an online social cognitive training program and conduct a pilot study to assess its feasibility within the current sample.

**Methods:**

36 participants (randomly assigned to 16 for the experimental group and 20 for the control group) were recruited for the pilot study and the experimental group completed 6 weeks of an online intervention program, while the control group was assigned to the waiting list. Autism Quotient, Depression Anxiety Stress -21, Patient Health Questionnaire -9, and Generalized Anxiety Questionnaire– 7 were used to assess mental health symptoms. Executive functions were evaluated using the Wisconsin Card Sorting Task, N-back Task, and Go/No-Go task. At the same time, social cognition was assessed through the Interpersonal Reactivity Index, The Eyes test, and the Self-Assessment Manikin.

**Results:**

2 x 2 mixed repeated measure ANOVA was conducted. The main effect of time and group was significant for mental health symptoms and working memory (*p* < 0.5). However, the interaction of time and group was not significant (*p* > .05). Communication and attention switching (two components of autistic traits) have significant impacts on the scores (*p* < 0.5).

**Conclusions:**

This pilot study demonstrated significant improvements in mental health scores, laying a solid foundation for further development and efficacy testing of the digital intervention program. This suggests that the intervention holds promise, particularly in targeting mental health improvements. Attention switching and communication contribute to the overall main effects highlighting the program’s potential in addressing key aspects of mental health.

**Disclosure of Interest:**

None Declared

